# Dermoscopic and Histopathological Features of Nonfamilial Multiple Trichoepitheliomas: A Case Report

**DOI:** 10.7759/cureus.116312

**Published:** 2026-09-15

**Authors:** Lalla Safia Echarif, Maryem Aboudourib, Abdeljalil Ouaaziz, Ouafa Hocar, Said Amal

**Affiliations:** 1 Department of Dermatology and Venereology, Mohammed VI University Hospital, Marrakech, MAR; 2 Biosciences and Health Laboratory, Faculty of Medicine and Pharmacy of Marrakech, Cadi Ayyad University, Marrakech, MAR; 3 Department of Pathology, Arrazi Hospital, Mohammed VI University Hospital, Marrakech, MAR; 4 Morphoscience Research Laboratory, Faculty of Medicine and Pharmacy of Marrakech, Cadi Ayyad University, Marrakech, MAR

**Keywords:** adnexal tumours, basal cell carcinoma, dermoscopy, histopathology, multiple trichoepithelioma, nonfamilial trichoepithelioma, trichoepithelioma

## Abstract

Multiple trichoepitheliomas are benign follicular tumours that usually develop during childhood or adolescence and progressively increase in number. We report the case of a 21-year-old man with multiple asymptomatic papules and nodules involving the face, scalp and upper trunk since the age of 13 years, with no family history of similar lesions. Dermoscopy showed prominent arborising telangiectasias, ivory-white structureless areas and milia-like cysts. Because several lesions raised concern for basal cell carcinoma (BCC), three lesions located on the face, scalp and back were biopsied. Histopathology of all three specimens showed a symmetrical, well-circumscribed dermal basaloid proliferation with keratin-filled lumina and a cellular, focally hyalinised stroma, without peripheral palisading, stromal retraction clefts or malignant proliferation. Genetic testing was unavailable, and the diagnosis of nonfamilial multiple trichoepitheliomas was based on the clinical presentation, absence of a family history and concordant histopathology. Topical rapamycin 1% provided limited cosmetic improvement after three months. Carbon dioxide laser therapy was considered, but was not financially accessible. The patient remains under regular dermatological follow-up.

## Introduction

Trichoepithelioma is a benign cutaneous adnexal neoplasm with follicular differentiation. It usually presents as asymptomatic, skin-coloured to pink papules or nodules, predominantly involving the face [1]. Multiple lesions generally begin during childhood or adolescence and increase progressively in size and number, sometimes extending to the scalp, neck and upper trunk. Extensive disease may result in considerable cosmetic and psychosocial burden [2]. Multiple trichoepitheliomas may occur in familial and nonfamilial forms. In familial disease, the presence of associated cylindromas or spiradenomas should raise suspicion for Brooke-Spiegler syndrome, one of the phenotypic expressions of germline CYLD alteration [1,2].

Dermoscopy can assist in the assessment of trichoepithelioma and serves as a complement to the histopathological evaluation. However, trichoepithelioma and basal cell carcinoma (BCC) often share clinical, dermoscopic and histopathological features, making the distinction difficult in some lesions [3]. We report a young man with extensive nonfamilial multiple trichoepitheliomas, focusing on their dermoscopic and histopathological characteristics, including BCC-like dermoscopic features that prompted biopsy of three anatomically distinct lesions.

## Case presentation

A 21-year-old man with no significant medical history presented with multiple asymptomatic skin-coloured papules and nodules that had first appeared at the age of 13 years and had slowly increased in size and number. There was no known family history of similar lesions.

Dermatological examination showed numerous translucent, pink-to-flesh-coloured papules and small nodules of variable size, flat-topped or dome-shaped, and confluent in some areas. They involved the nose, nasolabial folds, forehead, auricles, scalp and upper trunk, including the back (Figure 1). No cylindromas, spiradenomas or other associated adnexal tumours suggestive of a syndromic presentation were identified.

**Figure 1:**
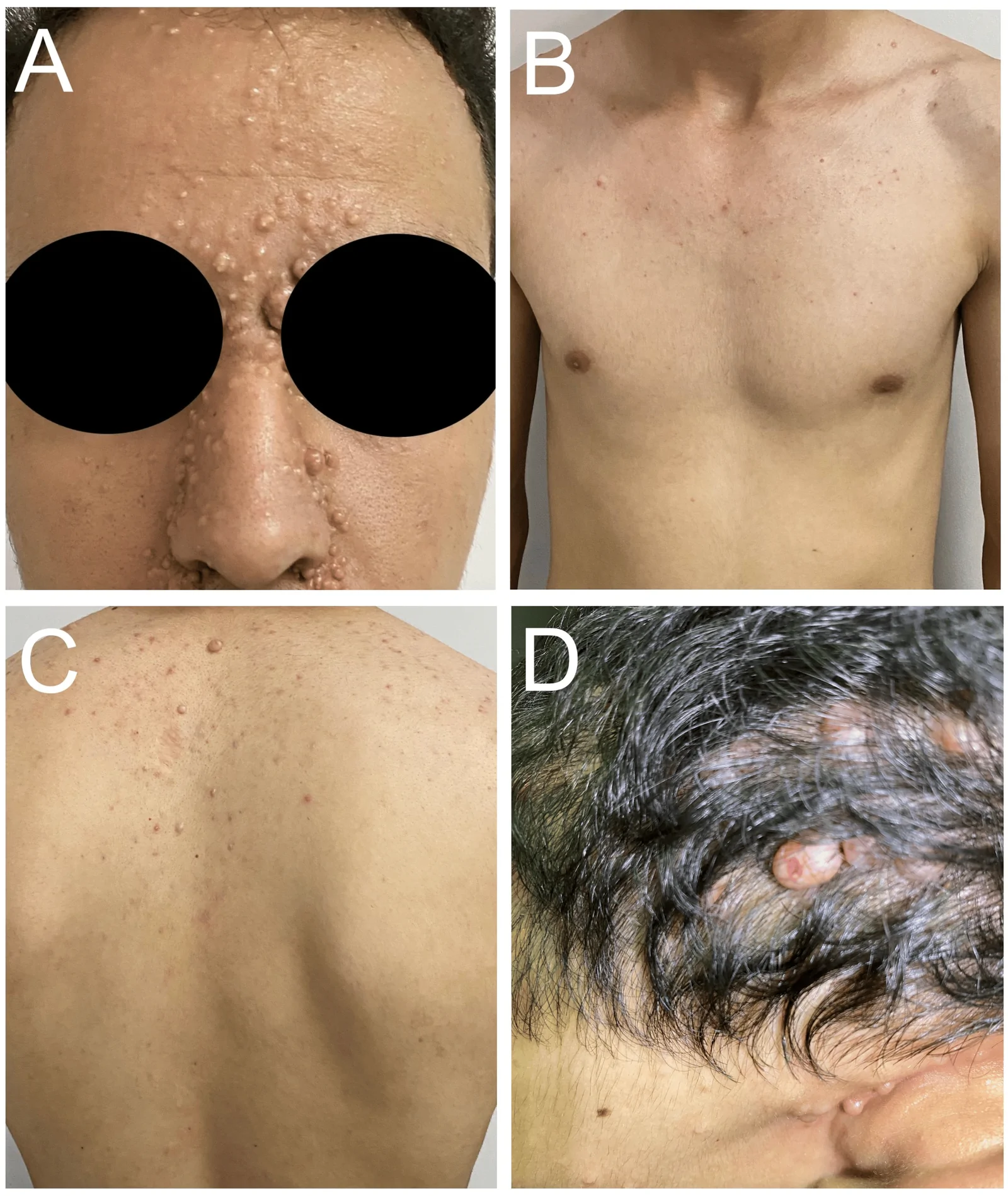
Overall clinical presentation of multiple trichoepitheliomas. (A) Multiple translucent, pink to flesh-coloured papules and nodules involving the central face. (B) Scattered papules over the anterior trunk. (C) Multiple papules involving the back. (D) Scalp involvement with several pink papules and nodules.

Dermoscopy of representative lesions showed prominent arborising telangiectasias, ivory-white structureless areas and milia-like cysts (Figure 2). Leaf-like structures and ovoid nests were not identified. Several lesions showed conspicuous vascular features raising concern for BCC; three of these lesions, located on the face, scalp and back, were therefore biopsied.

**Figure 2:**
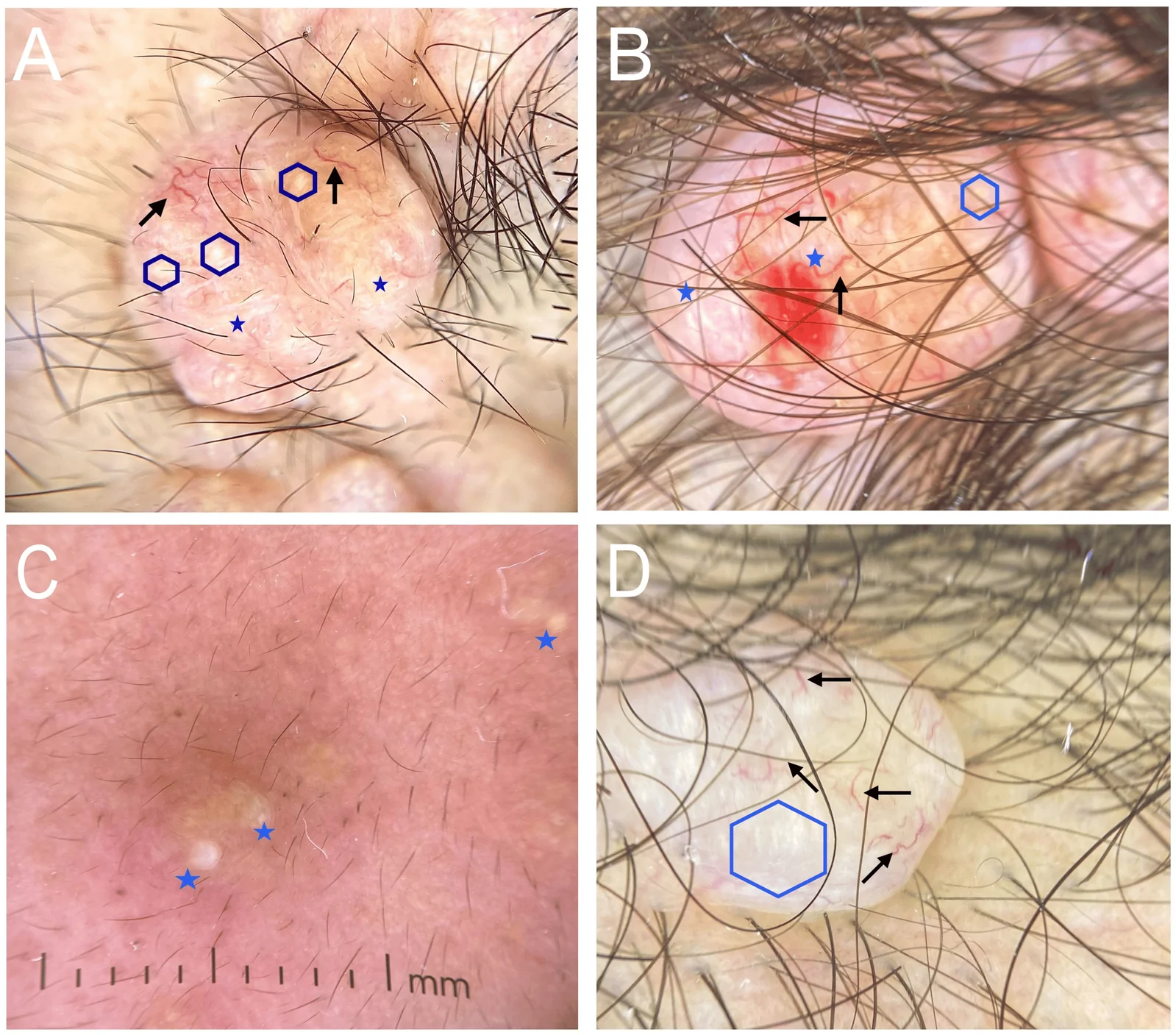
Dermoscopic findings in multiple trichoepitheliomas. (A) Arborising vessels (black arrows), ivory-white structureless areas (blue hexagons) and milia-like cysts (blue stars). (B) Arborising vessels (black arrows), ivory-white structureless areas (blue hexagons) and milia-like cysts (blue stars), with predominance of the vascular and whitish structureless components. (C) Predominantly milia-like cysts (blue stars). (D) Arborising vessels (black arrows) and ivory-white structureless areas (blue hexagons).

Histological examination of all three specimens showed a symmetrical, well-circumscribed but unencapsulated dermal adnexal proliferation, without connection to the epidermis, composed of small nests and lobules of basaloid epithelial cells. The stroma was cellular and focally hyalinised, and some tumour nests contained keratin-filled lumina. Peripheral palisading and stromal retraction clefts were absent. The tumour cells had round-to-oval, focally slightly enlarged and hyperchromatic nuclei, with occasional mitotic figures and moderately abundant basophilic cytoplasm. No malignant proliferation was identified (Figure 3).

**Figure 3:**
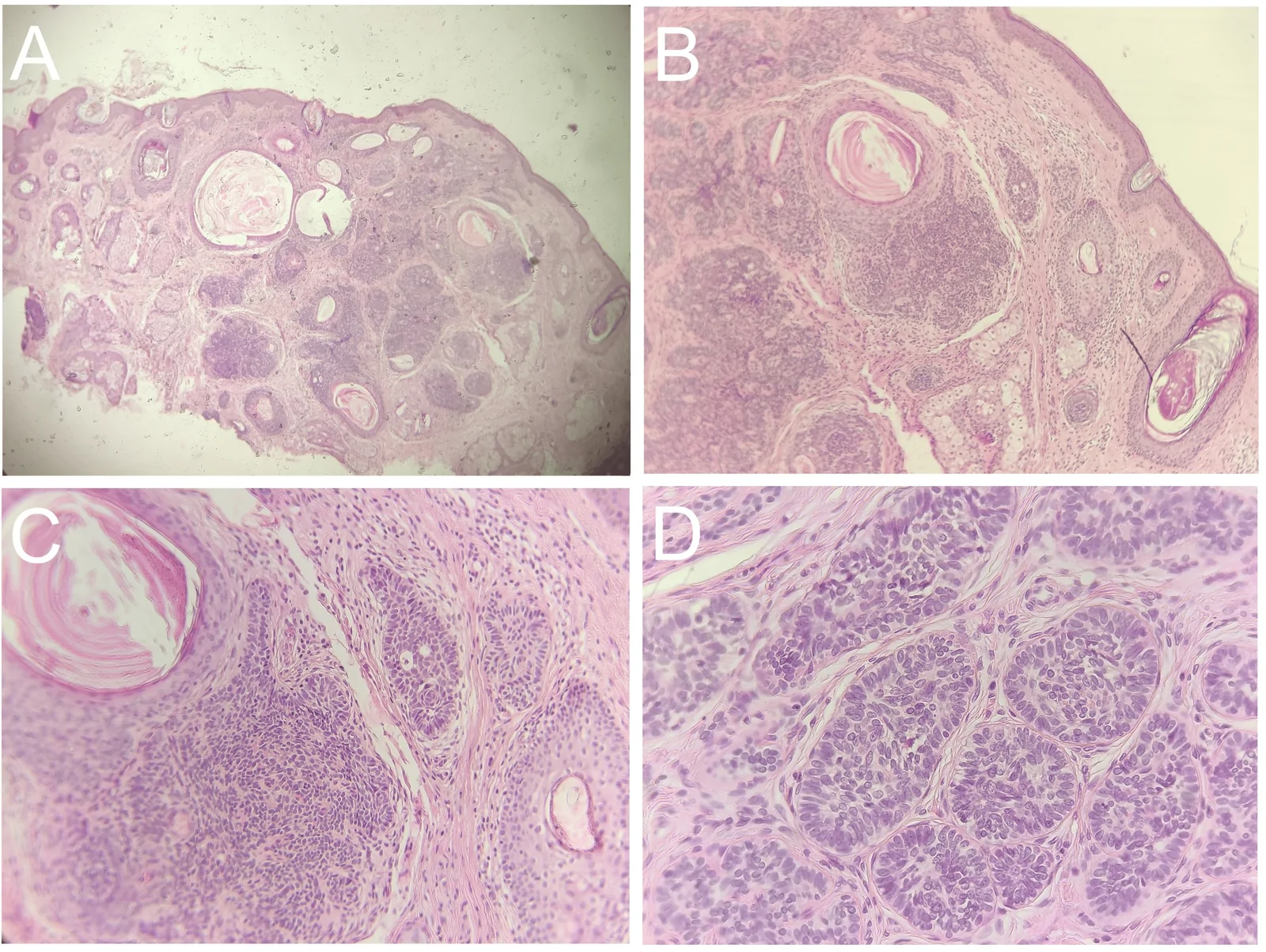
Histopathological findings of trichoepithelioma (H&E). (A) Low-power view showing the symmetrical, well-circumscribed dermal proliferation composed of multiple basaloid nests and lobules (×4). (B) Basaloid tumour lobules within the dermis, embedded in a cellular stroma (×10). (C) High-power view showing basaloid nests closely associated with the surrounding stroma and keratin-containing luminal structures (×20). (D) High-power view showing basaloid epithelial cells with round-to-oval, focally slightly enlarged and hyperchromatic nuclei (×40). H&E: hematoxylin and eosin

Genetic testing was unavailable. In the absence of similar lesions among relatives and with no associated adnexal tumours identified on examination, the presentation was considered clinically nonfamilial; the diagnosis of trichoepithelioma was based on the medical history, clinical presentation and concordant histopathological findings from the three sampled sites.

A compounded topical rapamycin 1% preparation was prescribed twice daily. After three months, the clinical improvement was insufficient to meet the patient’s cosmetic expectations, which remained his main concern. Carbon dioxide laser therapy was subsequently discussed but could not be pursued because of cost. The patient remains under regular dermatological follow-up to assess the evolution of existing lesions and identify new lesions or morphological changes that may warrant further biopsy.

## Discussion

Trichoepithelioma is classically divided into solitary, multiple, and desmoplastic forms. Multiple lesions typically develop early in life [1]. In their clinicopathological series, du Toit et al. reported multiple trichoepitheliomas that began at a mean age of 17.3 years and primarily affected the central face, though scalp and back involvement was also observed [4]. Our patient exhibited this early-onset pattern, with lesions appearing at 13 years of age and an unusually extensive distribution involving the face, auricles, scalp, and trunk.

The multiple form is classically familial [1], although nonfamilial presentations are well documented. Mandekou-Lefaki et al. described unilateral facial disease beginning in childhood in a woman without affected relatives [5]. Miotto and Romiti reported a more widespread distribution involving the face, external auditory canal, trunk and upper limbs; biopsies from facial and truncal lesions confirmed trichoepithelioma, while genetic testing was unavailable [6]. Our patient also had no family history and demonstrated an extensive nonfamilial presentation.

Multiple familial trichoepitheliomas are associated with germline mutations in the *CYLD* tumour-suppressor gene, although detectable mutations are not found in all clinically affected patients. Kazakov et al., in their clinicopathological and molecular series, identified germline *CYLD* mutations in 6 of 13 patients tested (46.2%) [7]. In our case, nonfamilial refers to the absence of affected relatives. Because genetic testing was unavailable, an underlying germline CYLD alteration could not be excluded.

The dermoscopic features of trichoepithelioma include arborising or branching vessels, milia-like cysts, rosettes, and blue-grey structures, typically observed on a whitish or translucent background [3,8]. Furthermore, a white-ivory background with bright white structures has been reported in nonfamilial multiple trichoepitheliomas [5]. In our patient, the main findings were prominent arborising vessels, milia-like cysts, and ivory-white structureless areas.

Syringoma and sebaceous hyperplasia also enter the differential of multiple facial papules [2], but the age at onset, progressive course, multiplicity and distribution of the lesions in our patient favoured multiple trichoepitheliomas [1,4]. Cylindroma remained relevant because it may coexist with trichoepithelioma in CYLD-associated disease [2] and can show branching vessels and white structureless areas on dermoscopy [9]. We identified no clinically distinct cylindromas or spiradenomas.

Several lesions, however, showed particularly prominent arborising vessels. This pattern can occur in trichoepithelioma and cylindroma [4,9], but in our patient it raised concern that an individual lesion might represent BCC. We specifically assessed for additional dermoscopic features of BCC, including blue-grey ovoid nests, multiple blue-grey globules, leaf-like structures and spoke-wheel areas [3], but none were identified. Despite their absence, the conspicuous vascular pattern kept BCC in the differential and led us to biopsy the most suspicious lesions.

Histologically, trichoepithelioma is characterised by a dermal proliferation of basaloid epithelial cells associated with keratinous or cystic differentiation and a closely related cellular or fibrous stroma [1]. Differentiation from BCC can be challenging, as both tumours may exhibit basaloid morphology and, in some trichoepitheliomas, peripheral palisading [3]. Symmetry and circumscription, keratinous differentiation, bland cytology and the relationship between the epithelial proliferation and its stroma favour trichoepithelioma, whereas greater cytological atypia, numerous mitotic figures, and prominent epithelial-stromal retraction are more characteristic of BCC [6]. In our patient, histology revealed a symmetrical, well-circumscribed dermal basaloid proliferation with keratin-containing lumina and a cellular, focally hyalinised stroma. Peripheral palisading and stromal retraction clefts were absent. Occasional mitotic figures were observed, but no malignant proliferation was present.

Immunohistochemistry may provide additional support when routine morphology remains equivocal. In the clinicopathological series by du Toit et al., six trichoepitheliomas showed features suggestive of BCC, whereas immunohistochemistry assisted the distinction in only one case; the authors considered routine histology with clinical correlation the diagnostic standard [4]. Stromal CD34 expression and differences in BerEP4 staining have also been used as ancillary findings in difficult cases [10]. Immunohistochemistry was not performed; the diagnosis was established on morphological grounds.

Dermoscopy and histopathology provided complementary findings, although direct structure-to-structure correlation was limited. Milia-like cysts have previously been correlated with keratin cysts on histopathological examination in multiple trichoepitheliomas [11]. White structureless areas in adnexal tumours have also been associated with fibrous stroma [9]. In our patient, however, histology showed keratin-containing lumina but no horn cysts, so the correlation with the dermoscopic milia-like cysts could not be demonstrated. Ivory-white structureless areas were also present dermoscopically and the stroma was focally hyalinised, but a direct relationship between these findings could not be established. No specific histological correlate was identified for the prominent vascular pattern.

Treatment of multiple trichoepitheliomas is particularly difficult when lesions are numerous or involve cosmetically sensitive areas. Surgical excision, electrosurgery, radiofrequency ablation, dermabrasion, cryotherapy and several laser modalities have been used. However, repeated procedures may be limited by scarring, recurrence, incomplete clearance and the continuing development of lesions. Pharmacological treatment remains supported mainly by small series and case reports. In the review by Lobo et al., none of 16 pharmacologically treated patients achieved complete clearance; 15 had a partial response and one had no response [2].

These data informed our choice of topical rapamycin. Dreyfus et al. treated five patients with multiple familial trichoepitheliomas using topical rapamycin 1% twice daily. At five months, four patients reported improvement, whereas one did not respond [12]. Our patient received the same concentration and frequency. After three months, the response remained insufficient to meet his cosmetic expectations. We then considered carbon dioxide laser; this technique has been used for multiple trichoepitheliomas, including in the largest published laser series [13], but it was not financially accessible to our patient.

Trichoepithelioma generally follows a benign course, although transformation to BCC or trichoblastic carcinoma has been reported [1,2,6]. Rapid growth, ulceration or bleeding in a pre-existing lesion should prompt reassessment [1]. Dermoscopy may also be useful during follow-up to identify a lesion that develops a pattern different from the surrounding trichoepitheliomas and warrants histological reassessment [3,11].

## Conclusions

Genetic testing was not available in our patient. The presentation was considered clinically nonfamilial because no affected relatives were identified, while the diagnosis of trichoepithelioma was based on the clinical distribution of the lesions, the dermoscopic pattern and concordant histopathology. Dermoscopy was particularly helpful in identifying lesions with a vascular pattern that remained suspicious for BCC and warranted biopsy.

Treatment remains challenging. Three months of topical rapamycin 1% did not provide the cosmetic improvement expected by the patient, while carbon dioxide laser therapy was not financially accessible. During follow-up, dermoscopy may help identify lesions that develop a different appearance and require histological reassessment.
